# Endocervical and vaginal microbiota in South African adolescents with asymptomatic *Chlamydia trachomatis* infection

**DOI:** 10.1038/s41598-018-29320-x

**Published:** 2018-07-23

**Authors:** Christina Balle, Katie Lennard, Smritee Dabee, Shaun L. Barnabas, Shameem Z. Jaumdally, Melanie A. Gasper, Venessa Maseko, Zizipho Z. A. Mbulawa, Anna-Lise Williamson, Linda-Gail Bekker, David A. Lewis, Jo-Ann S. Passmore, Heather B. Jaspan

**Affiliations:** 1 Institute of Infectious Disease and Molecular Medicine & Division of Immunology, Department of Pathology, University of Cape Town, Cape Town, South Africa; 2 Computational Biology Division, Department of Integrative Biomedical Sciences, University of Cape Town, Cape Town, South Africa; 3 Institute of Institute of Infectious Disease and Molecular Medicine & Division of Medical Virology, Department of Pathology, University of Cape Town, Cape Town, South Africa; 4 Desmond Tutu HIV Centre, University of Cape Town, Cape Town, South Africa; 5 Seattle Children’s Research Institute, Seattle, WA USA; 6 National Institute for Communicable Diseases, Sandringham, Johannesburg, South Africa; 7 Center for HIV and STIs, National Institute for Communicable Disease, National Health Laboratory Service, Johannesburg, South Africa; 8 SAMRC-UCT Clinical Gynaecological Cancer Research Centre, University of Cape Town, Cape Town, South Africa; 9 Western Sydney Sexual Health Centre, Parramatta, Australia; 10 Marie Bashir Institute for Infectious Diseases and Biosecurity & Sydney Medical School-Westmead, University of Sydney, Sydney, Australia; 11 DST-NRF CAPRISA Centre of Excellence in HIV Prevention, Durban, South Africa; 12 National Health Laboratory Service Cape Town, Cape Town, South Africa; 13 University of Washington Department of Pediatrics and Global Health, Seattle, WA USA

## Abstract

Adolescent girls and young women represent a key risk group for sexually transmitted infections (STIs). The vaginal microbiota is thought to play an important role in susceptibility to STIs such as *Chlamydia trachomatis*. We compared the microbiota of the lateral vaginal wall and endocervix, and assessed associations with *C*. *trachomatis* infection in South African adolescents. The endocervical and vaginal lateral wall microbiota were characterized by amplifying and sequencing the V4 region of the 16S rRNA gene and *C*. *trachomatis* diagnosed using molecular methods. Of the 72 girls included, 30 had asymptomatic *C*. *trachomatis* infections. Three major vaginal community types were identified; one *Lactobacillus crispatus*, *one L*. *iners* and one diverse, *Gardnerella vaginalis* dominant. The microbiota of the endocervix was significantly different from that of the lateral wall in terms of diversity. There were many differentially abundant taxa between the endocervix and lateral vaginal wall, including *Achromobacter spanius* and *Enterococcus faecium*. Women with *C*. *trachomatis* had higher relative abundance of *G*. *vaginalis* and other anaerobes. In this African adolescent cohort, significant differences between the lateral vaginal wall and endocervical microbiota diversity and composition were evident, although neither were strongly associated with *C*. *trachomatis* infection.

## Introduction

*Chlamydia trachomatis* is an obligate intracellular bacterium and the leading cause of bacterial sexually transmitted infections (STIs) worldwide with young women being particularly at risk^1^. Up to 80% of *C*. *trachomatis* infections in women are asymptomatic. If not treated, *C*. *trachomatis* can cause serious complications such as pelvic inflammatory disease, ectopic pregnancy and infertility. Bacterial vaginosis (BV) is a condition characterized by the replacement of *Lactobacillus* species in the female genital tract (FGT) with various anaerobic and facultative bacteria, including *Gardnerella vaginalis*, *Atopobium vaginae*, *Megasphaera*, *Prevotella*, and BV-associated bacteria (BVAB) 1–3. Molecular-based studies of healthy, reproductive-age women have described a distinct group of common vaginal bacterial community types, which vary according to population^2,3^. In general, low-diversity communities dominated by a single *Lactobacillus* species (*Lactobacillus crispatus*, *L*. *gasseri*, *L*. *iners*, or *L*. *jensenii*) and one to two high-diversity communities, comprised of a mixture of anaerobic bacteria associated with BV, are described^3–6^. *Lactobacillus* species are thought to protect the lower genital tract from invading pathogenic microorganisms by competing for nutrients, secretion of bacteriostatic and microbicidal compounds and by lowering the pH (<4.5) through lactic acid production.

Several studies have demonstrated a positive association between BV and the incidence of STIs, including *C*. *trachomatis*, *Neisseria gonorrhoeae* and Human Immunodeficiency Virus (HIV)^7–10^. Recent molecular studies have shown that women with *C*. *trachomatis* infection are more likely to have *L*. *iners*-dominated or dysbiotic vaginal communities compared to those without^11–13^. Although less well studied, the composition of the endocervical microbiota may be of greater importance when looking at the relationship between commensal bacteria and STIs, as the cervix is the main site of infection for several pathogens including *N*. *gonorrhoeae* and *C*. *trachomatis*^14^. Whereas the vagina has stratified squamous epithelium, *C*. *trachomatis* infects the columnar epithelial cells of the cervix. The cervical transformation zone is enriched with T cells and antigen-presenting cells compared to the vagina^15^ and differing immune and physiological environments (including different oxygen levels) could impact the microbial composition in different regions of the lower FGT. Molecular studies characterizing bacterial communities at different anatomical sites throughout the FGT have generated varying results with regards to microbial homogeneity^16–18^. Distinct microbial communities in cervix differing from that of the vagina have been described^19^. It is therefore of great importance to characterize endocervical microbial communities when studying the role that commensal bacteria play in *C*. *trachomatis* infection. To our knowledge, few molecular studies have evaluated the relationship between prevalent *C*. *trachomatis* infections and the composition of cervicovaginal microbiota and none have focused on African adolescents, a group at extreme risk for STIs. Therefore, we compared the endocervical and vaginal microbiota and evaluated their association with *C*. *trachomatis* infection in young South African females from a high risk community.

## Results

### Study population characteristics

Of the 154 lateral vaginal wall and endocervical samples taken from 77 participants, 149 samples passed the sequencing and quality control (≥5000 reads/sample, 3 endocervical and 2 lateral wall samples failed). Downstream analyses were conducted on 72 participants for whom both the lateral wall and matched endocervical samples passed quality control measures. Of those 72 participants, 42 (58%) were *C*. *trachomatis* negative and 30 (42%) *C*. *trachomatis* positive. None of the 30 women infected with *C*. *trachomatis* had LGV serovar infections. Nugent scores were available for all 72 participants. Nearly half of the participants (49%, n = 35) were BV positive (Nugent score 7–10), 5% were BV intermediate (Nugent score 4–6, n = 4) and the remaining 46% were BV negative (Nugent score 0–3, n = 33) (Table 1). The *C*. *trachomatis* positive and negative participants were similar in terms of most demographic and behavioral characteristics including age, body mass index (BMI), intravaginal practices and hormonal contraceptive use. Participants with BV were almost twice as likely to be infected with *C*. *trachomatis* compared to BV negative participants, but these observations were not statistically significant (OR = 1.8; 95% CI: 0.71–4.98, p = 0.21).

**Table 1:** Study population characteristics.

	*C*. *trachomatis* negative (n = 42)(58%)	*C*. *trachomatis* positive (n = 30)(42%)	P-value*
Age, years (median)	18.5	18	0.80
**Hormonal contraception**			0.73
*Injectable*	36 (86%)	27 (90%)	
*Non-injectable*	6 (14%)	3 (10%)	
*No (none)*	0 (0%)	0 (0%)	
BMI (median)^a^	25.39	25.15	0.35
**Intra-vaginal practices** ^**b**^			
*Douching*	3 (8%)	0 (0%)	0.31
*Washing w*. *water*	31 (97%)	26 (93%)	0.91
*Washing w*. *soap*	22 (67%)	17 (63%)	0.98
Lifetime # partners (median)	3	2	0.20
Multiple partners^c^	22 (61%)	10 (36%)	0.32
Regular condom use^d^	25 (76%)	15 (56%)	0.17
**BV prevalence**			0.10
*BV positive*	17 (40%)	18 (60%)	
*BV intermediate*	4 (10%)	0 (0%)	
*BV negative*	21 (50%)	12 (40%)	
Endocervix Alpha Diversity (Shannon, median)	1.1	1.8	0.28
Vaginal pH (median)	4.7	4.7	0.77
*N*. *gonorrhea* positive	2 (0.5%)	7 (23%)	0.02
HPV positive^e^	17 (49%)	15 (75%)	0.09

*Chi-squared test (Fisher’s exact test when expected values < 5) for the assessment of association of frequency among groups and the Mann–Whiney U-test for comparison of medians. Unpaired t-test for alpha diversity.

BV; bacterial vaginosis, STI; sexually transmitted infection, OCP; oral contraceptive pills.

^a^participants had missing information.

^b^11 participants had missing information.

^c^15 participants had missing information.

^d^12 participants had missing information.

^e^17 participants had missing information.

Adolescents infected with *C*. *trachomatis* were less likely to report multiple sexual partners and regular condom use (over the past year) and reported fewer lifetime sexual partners than *C*. *trachomatis* negative participants; although not significantly. *C*. *trachomatis* positive participants were, however, significantly more likely to be co-infected with *N*. *gonorrhea* than *C*. *trachomatis* negative participants (23% versus 0.5%, p = 0.03, Table 1).

### Comparison of vaginal and endocervical microbial composition

**Figure 1:**
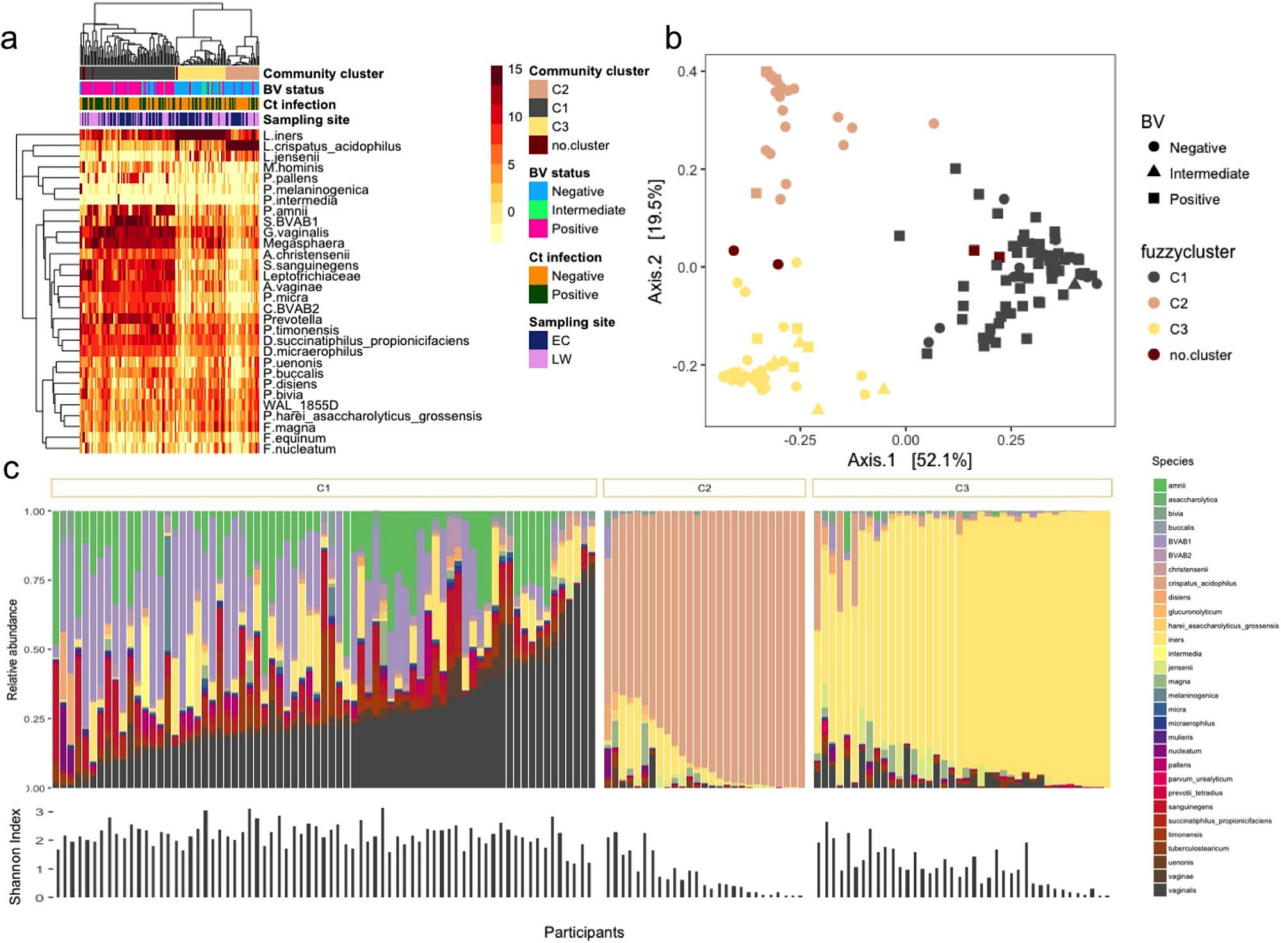
Composition of vaginal lateral wall and endocervical microbiota. (**a**) Heat map of the 30 most abundant taxa (rows) identified by 16S rRNA microbiome profiling using unsupervised hierarchical clustering with weighted UniFrac distances in all samples (columns). Unsupervised hierarchical clustering of 144 matched endocervial and vaginal lateral wall samples from 72 participants. The dendrogram was generated using average linkage clustering with weighted UniFrac distance, based on the relative abundance of taxa (merged at lowest taxonomic level) in each sample. Log2-transformed standardized read counts are illustrated by the colour key. Annotation bars above the heatmap depict community type cluster (top bar), BV status based on Nugent scoring (upper middle bar), *C*. *trachomatis* (Ct) infection status (lower middle bar) and anatomical sampling site (bottom bar). Samples that did not meet the minimum probability of ≥60% of belonging to any of the three clusters were excluded from downstream analyses (n = 4, “no.cluster” in figure). (**b**) Barplot of the 30 top most abundant taxa identified by 16S rRNA microbiome profiling. Samples are grouped by microbial compositional subtype (C1, C2, C3) established using Fuzzy clustering with weighted UniFrac distances and ordered based on the abundance of the most dominant species in each community type (*G*. *vaginalis*, *L*. *crispatus* and *L*. *iners*, respectively). Samples that did not meet the minimum probability of ≥60% of belonging to any of tree clusters (n = 4) were excluded from the figure. Shannon diversity Index for each sample is depicted below the barplot. (**c**) Principal Coordinates Analysis (PCoA) of samples colored by compositional subtypes generated using Fuzzy clustering with weighted UniFrac distances. Samples are colored by compositional subtype (C1, C2, C3), with BV status displayed as shapes. Samples that did not meet the minimum probability of ≥60% of belonging to any of these clusters were excluded from downstream analyses (n = 4, “no.cluster” in figure).

A total of 365 OTUs were generated and allocated to 332 different taxa. Using fuzzy clustering with optimal k and data from both sample sites (lateral wall and endocervix), we identified three major community types (C1–3, Fig. 1) in agreement with previous data from this cohort^20^. These community types consisted either predominantly of diverse anaerobic bacteria (C1, n = 73; 51%), *L*. *crispatus* (C2, n = 27; 19%) or *L*. *iners* (C3, n = 40; 28%), respectively. The most abundant species in C1 was *G*. *vaginalis* followed by BVAB1, BVAB2, *Prevotella amnii*, *L*. *iners*, *Sneathia sanguinegens*, *Prevotella timonensis*, *A*. *vaginae*, *Dialister* spp., *Prevotella pallens* and *Megasphaera* spp. The majority of participants with a C1 community type were BV positive according to Nugent scoring (82%) whereas participants with C2 and C3 community types were most commonly BV negative (89% and 73%, respectively, Fig. 1a–c).

**Figure 2:**
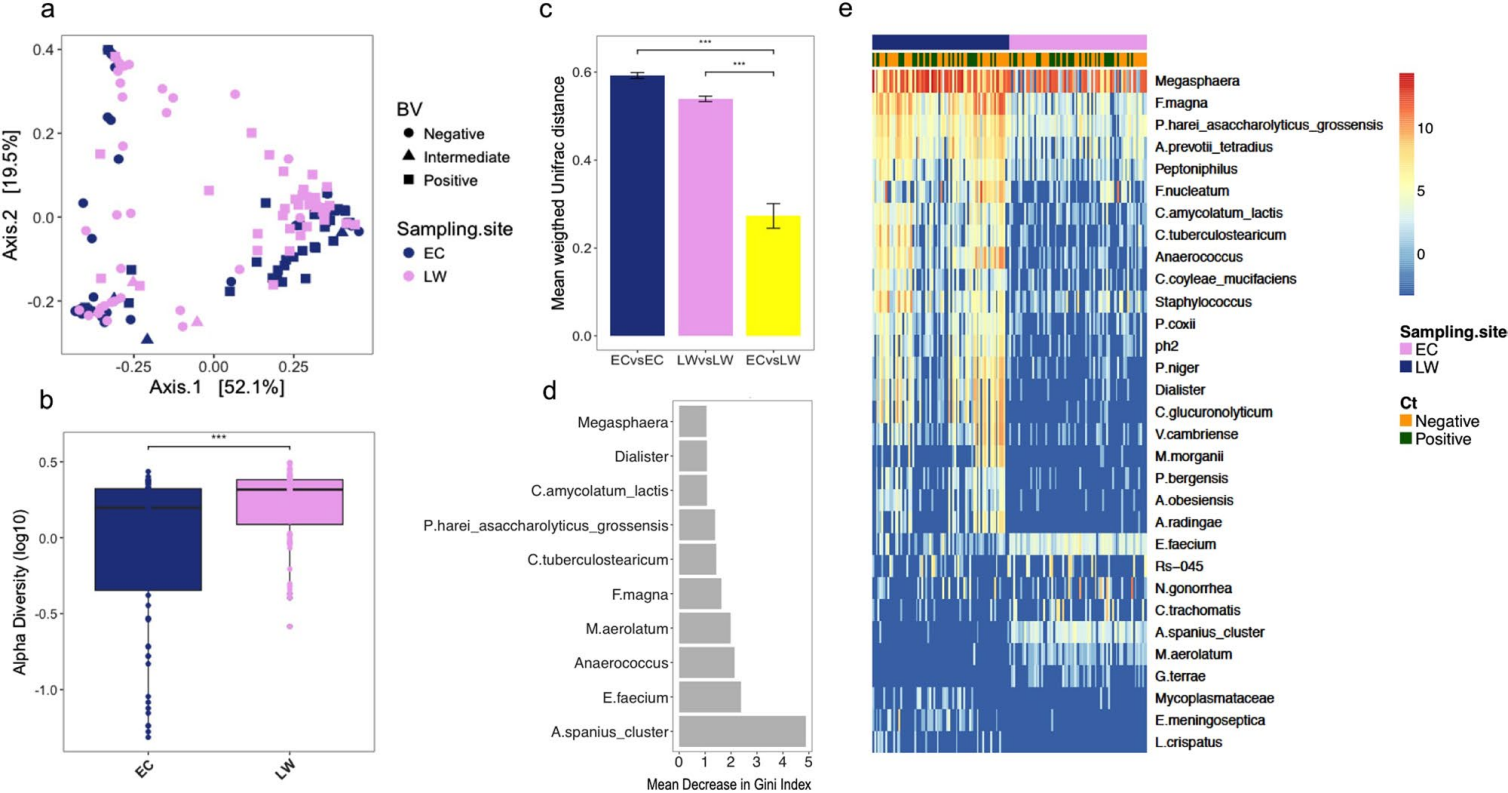
Taxa significantly different between vaginal lateral wall versus endocervical microbiota. (**a**) Principal Coordinates Analysis (PCoA) of the vaginal and endocervical microbiota using weighted UniFrac distances. Individual samples are colored by anatomical sampling site (endocervical:EC and lateral vaginal wall:LW) with BV status displayed as shapes. (**b**) Boxplot depicting the alpha diversity of the vaginal lateral wall (LW) and endocervical (EC) microbiota. (**c**) Taxa significantly differentially abundant and/or frequent by anatomical site category by metagenomeSeq (FDR ≤0.05, coefficient ≥1.25, taxa present in ≥20% of samples in at least one of the two groups being compared). Unsupervised clustering of samples (columns) by Bray-Curtis distance; heat map scale: log2-transformed standardized counts. (**d**) The top ten most influential taxa by random forests analysis. The x-axis indicates the mean decrease in Gini Index (length of bar represents predictive ability of each taxon). (**e**) Barplot depicting within-subject pair-wise distances (weigthed UniFrac) between sampling sites (ECvsLW) and the mean between-subject pair-wise distances (weighted UniFrac) between either endocervical samples (ECvsEC) or lateral wall samples (LWvsLW).

To evaluate the differences in overall bacterial community composition between sampling sites, principal coordinates analysis (PCoA) using weighted UniFrac distances was performed (Fig. 1b). The samples clearly grouped into three distinct clusters corresponding to the three community types described above (Fig. 1b). However, within the three clusters the samples separated according to sampling site (Fig. 2a). (Adonis p = 0.002). Furthermore, lateral wall samples had significantly higher species richness and alpha diversity compared to endocervical samples (p < 0.001, Fig. 2b). Yet, for 82% of the participants, the lateral wall and endocervical samples were assigned to the same community cluster and the within-participant variability was significantly less than the variability of samples from same site between participants based on mean weighted Unifrac distances (p < 0.001 for endocervical and lateral wall, Student’s paired t-test, Fig. 2e).

In order to identify differentially abundant bacterial species by anatomical sites, metagenomeSeq analysis was applied. The relative abundances of 31 taxa (OTUs merged at lowest taxonomic level) were significantly different between endocervical and lateral wall samples (Fig. 2c). These included *Achromobacter spanius_cluster*, *Gordonia terrae*, *Methylobacterium aerolatum*, *Enterococcus faecium*, *C*. *trachomatis* and *N*. *gonorrhea*, which were all more abundant in the endocervical samples, and *Peptoniphilus* spp., *Anaerococcus* spp., *Corynebacterium* ssp., *Megasphaera*, *Morganella morganii*, *Finegoldia magna*, *Elizabethkingia meningoseptica*, *Staphylococcus*, *Actinomyces radingae*, *Prevotella bergensis*, *Dialister*, *Fusobacterium nucleatum*, *Peptococcus niger*, *Varibaculum cambriense* and *Lactobacillus crispatus* which were more abundant in lateral wall samples. Random forest analysis was additionally used to identify species predictive of sampling site. In concordance with the metagenomeSeq results, the most influencial taxa in differentiating endocervical from lateral wall included *A*. *spanius_cluster*, *E*. *faecium*, *M*. *aerolatum*, *Peptoniphilus harei_asaccharolyticus_grossensis* and *Megasphaera* which were more prominent in endocervical samples, and *Anaerococcus*, *F*. *magna*, *Dialister* and *Corynebacterium* ssp., which were more common in lateral wall samples (Fig. 2d, AUC = 0.98, PPV = 0.96, NPV = 0.90 for the test set and a validation predicted error rate of 6.25%).

### Relationship between genital tract microbiota with *C. trachomatis* infection

The association between *C*. *trachomatis* infection and microbiota was considered separately for lateral wall and endocervical samples. The alpha diversity of endocervical microbiota tended to be higher in *C*. *trachomatis* positive participants compared to *C*. *trachomatis* negative participants, although not significantly (1.8 versus 1.1, p = 0.28, Table 1); this was also the case in lateral wall samples (p = 0.32, data not shown). In univariate analysis, participants with a community type dominated by diverse anaerobic bacteria (C1) or *L*. *iners* (C3) were more likely to be infected with *C*. *trachomatis* compared to those having an *L*. *crispatus*-dominated community, although not significantly so (OR = 2.98; 95% confidence interval: 0.76–15.0 and OR = 2.50; 95% CI: 0.56–13.6, respectively). None of the study participant demographic or behavioral factors listed in Table 1 were associated with *C*. *trachomatis* in univariate or multivariate analyses.

**Figure 3:**
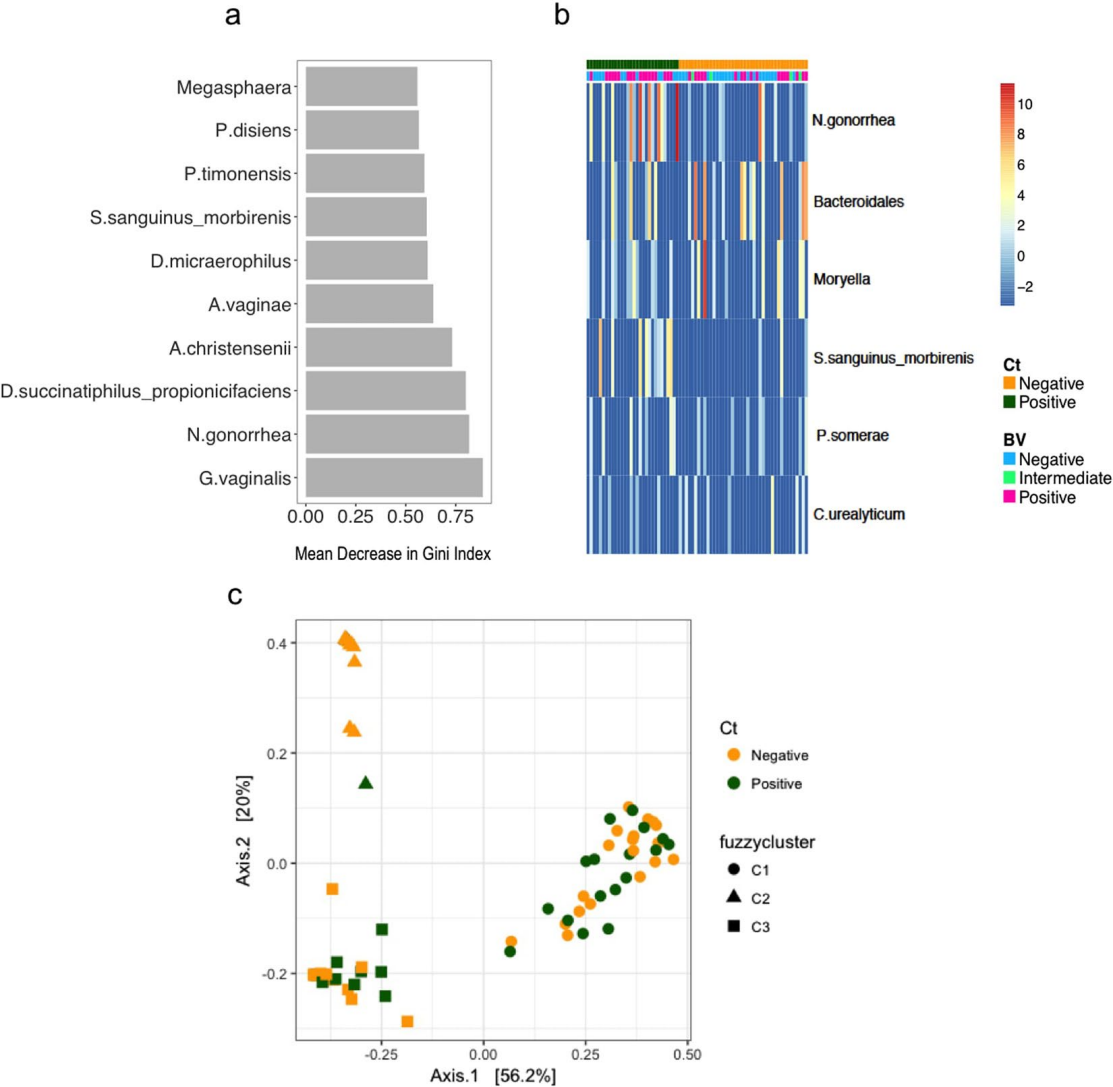
Taxa significantly different in *C*. *trachomatis* infected versus uninfected individuals in the endocervical microbiota. (**a**) Taxa significantly differentially abundant and/or frequent by *C*. *trachomatis* category in the endocervical (EC) microbiota by metagenomeSeq (FDR ≤0.05, coefficient ≥1.25, taxa present in ≥20% of samples in at least one of the two groups being compared). Unsupervised clustering of samples (columns) by Bray-Curtis distance; heat map scale: log2-transformed standardized counts. The OTU assigned *C*. *trachomatis* taxonomy was excluded for this analysis. (**b**) The top 20 most influential taxa by random forests analysis. The x-axis indicates the mean decrease in Gini Index (length of bar represents predictive ability of each taxon), where a larger index indicates greater predictive power. Taxa that were significantly differentially abundant and/or frequent in *C*. *trachomatis* infected versus uninfected individuals (FDR ≤0.05, coefficient ≥1.25, taxa present in ≥20% of samples in at least one of the two groups being compared); hierarchical clustering (Bray-curtis distance); heat map scale: log2-transformed standardized counts. The OTU assigned *C*. *trachomatis* taxonomy was excluded for this analysis. (**c**) Principal Coordinates Analysis (PCoA) of the endocervical microbiota using weighted UniFrac distances. Individual samples are colored by *C*. *trachomatis* infection status with fuzzycluster displayed as shapes.

On the other hand, differential abundance testing (using metagenomeSeq) revealed many significantly differentially abundant cervical taxa between *C*. *trachomatis* positive versus negative women, including as expected higher relative abundance of *N*. *gonorrhea*, but also Rs-045, *Sutterella sanguinus_morbirenis*, *Porphyromonas somerae* and *Corynebacterium urealyticum* (Fig. 3b). Using random forest analysis, these same species, as well as *G*. *vaginalis*, *Aerococcus christensenii*, *Dialister* spp., *Megasphaera*, *A*. *vaginae* and *Prevotella disiens* were found to be predictive of *C*. *trachomatis* infection (Fig. 3b). However, the predictive model had an AUC of 0.46 and predicted error rate of 54.17%. Many of these same genera were also predictive of *C*. *trachomatis* infection status in lateral wall samples, with *Prevotella* spp. and *Megasphaera* being the strongest predictors (Supplementary Figure 1); however this predictive model was very weak with an predicted error rate of 66.67% and AUC of 0.29 for the test set. In concordance with this, no overall differences in microbiota composition (β-diversity) were found between *C*. *trachomatis* positive and *C*. *trachomatis* negative participants (Fig. 3c, Adonis p = 0.27 and p = 0.58 for endocervical and lateral wall, respectively).

## Discussion

Adolescent girls and young women in sub-Saharan Africa are at high risk for STIs and frequently have vaginal dysbiosis^20^. To our knowledge, this is the first study to describe the relationship between genital tract microbial communities and *C*. *trachomatis* in adolescents. Adolescence is the period of greatest risk for STI acquisition. Furthermore, few studies have examined this relationship in African women, who have very different vaginal microbiota than their European counterparts. Finally, most if not all, studies interrogating the relationship between *C*. *trachomatis* and genital tract microbiota have focused only the vagina, which is not the site of *C. trachomatis* infection. Here, we find significant differences between endocervical and vaginal microbiota, and identify associations between cervical bacterial taxa and *C*. *trachomatis* infection in African adolescents.

Although the overall bacterial community compositions were found to be more similar between the endocervix and vagina within each participant than between participants, we found significantly different β-diversity between endocervical and vaginal samples within clusters. Furthermore, consistent with the results of others^21^ we found the within sample diversity in the vagina to be higher than in the ectocervix. Although previous studies have suggested that the microbiota in different anatomical sites within the FGT of adults is similar in composition^16–18^, we identified multiple taxa that were significantly more abundant in the vagina. These included anaerobes, such as *Peptoniphilus*, *Anaerococcus* and *Fusobacterium* spp. The endocervix also had significantly more *E*. *faecium*, the source of which is likely the gastrointestinal tract. These findings are surprising, since the endocervix is further from the external (aerobic) environment and the rectum, than the vagina. In Chinese women^19^, *L*. *crispatus* was notably more abundant in the vagina compared to the endocervix, which may be due to epithelial cell characteristics. *L*. *crispatus* expresses an adhesion molecule which mediates adherence to stratified squamous epithelium (found in the vagina) but not to columnar epithelium which constitutes the cervical epithelium^22^. Therefore, factors other than geography likely play a role in microbial ecosystems in the FGT. Since adolescence is a time of change in the cervix, it is possible that the differences we see here are unique to this age group. Alternatively, differences in sample collection technique between lateral vaginal wall and endocervical swabs may have occurred.

No studies have explored the relationship between specific taxa in the endocervix and *C*. *trachomatis* in adolescents. Our findings suggest the endocervical microbiota diversity is not grossly altered in women with *C*. *trachomatis*, although the study was small. However, we found some taxa differentially abundant between *C*. *trachomatis* infected and uninfected women, including *G*. *vaginalis*, *Megasphaera*, *A*. *vaginae*, *Dialister ssp*., *Prevotella spp*. and *Megasphaera*, all BV-associated bacteria. Several studies have reported an increased risk of *C*. *trachomatis* infection among women with BV^2,5,23^. In a longitudinal study among Dutch women, microbiota dominated by *L*. *iners* was an independent risk factor for later *C*. *trachomatis* acquisition^12^, and *L*. *crispatus* may have been protective^11–14^. The correlation between lactobacilli and vaginal health is often attributed to the production of lactic acid resulting in low vaginal pH. Importantly, *L*. *crispatus* produces significantly more D-lactate than *L*. *iners*, and this difference has been shown to be important in trapping of pathogens^24^. Indeed, *L*. *crispatus* has been shown to inhibit growth, adhesion and infectivity of *C*. *trachomatis in vitro*, attributed to the production of D-lactate^25–27^. Of note, *L*. *iners* is far more prevalent than other lactobacilli in African women, often co-exists with BV^9,20^, and may be one of the reasons why women of African descent are at high risk for STIs.

One limitation of this study is its cross-sectional nature, which precludes definitive conclusions regarding whether specific taxa directly increase risk of *C*. *trachomatis* infection. Prospective longitudinal cohort studies will allow comparison of microbial communities prior to and after infection with *C*. *trachomatis*, which will allow us to make conclusions regarding the role of microbiota in modifying *C*. *trachomatis* risk in adolescents. None-the-less, the potential for altered functional profiles of *C*. *trachomatis* susceptible bacterial communities between different anatomical sites exists, despite overall compositional similarities between women with and without *C*. *trachomatis* infections.

## Methods

### Study participants and sample collection

The study population consisted of 149 16–22-year-old women from a low income, high population density community in Cape Town, South Africa. Participants were enrolled in the Women’s Initiative in Sexual Health (WISH) study between November 2013 and December 2014. Detailed procedures and characteristics of the cohort have previously been described^28^. Approval for the study was obtained from the Human Research Ethics Committee of the University of Cape Town and all study procedures were conducted in accordance with the International Conference on Harmonization (ICH) and the South African Good Clinical Practice Guidelines^29^. All participants 18 years or older provided informed consent, while informed parental consent and participant assent were obtained for those younger than 18 years. Females were enrolled if they were HIV-negative, in good health, not pregnant or menstruating at the time of sampling, if they had not had unprotected sex or douched in the last 48 hours, nor taken antibiotics in the prior two weeks. The women were followed longitudinally every two months if they were using Norethisterone enanthate (NET-EN) injectable contraceptive, combined oral contraceptives or barrier contraception only, or every three months if they were using depot medroxyprogesterone acetate (DMPA) injectable contraceptive, for a total of three visits. Study visits were scheduled two weeks after injection for participants on injectable contraceptives, or otherwise during the luteal phase of their menstrual cycles (between day 14–28) if they were not using hormonal contraceptives or if they were using oral contraceptives. Prior to any specimen collection, an HIV rapid test (Alere Determine™ HIV-1/2 Ag/Ab Combo, Alere, Waltham, MA), a pregnancy test (U-test Pregnancy strip, Humor Diagnostica, Pretoria, South Africa) and a general physical examination was performed. At each visit, vulvovaginal swabs for STI testing and Nugent scoring, as well as swabs from the lateral vaginal wall and endocervix for microbiome analyses, were obtained.

### STI and BV testing

Vulvovaginal swabs were tested with a variety of in-house multiplex and commercial PCR assays (*Chlamydia trachomatis*, *Neisseria gonorrhoeae*, *Trichomonas vaginalis*, *Mycoplasma genitalium*, herpes simplex virus type 1 (HSV-1) and HSV-2, *Haemophilus ducreyi*, *Treponema pallidum*, for positive *C*. *trachomatis* DNA extracts, additional testing for *Lymphogranuloma venereum-*associated serovars) as previously described^21^. Chlamydial infections were further differentiated into lymphogranuloma venereum (LGV)-associated serovars or non-LGV serovars, using an LGV-specific PCR. Blood was obtained for HIV rapid testing and HSV-2 serology. Endocervical swabs were collected for human papillomavirus (HPV) detection and genotyping by Roche Linear Array^30^. Posterior fornix swabs were collected for Nugent scoring to classify samples as BV negative (Nugent 0–3), intermediate (Nugent 4–6) or positive (Nugent 7–10); and vaginal pH was measured using colour-fixed indicator strips (Macherey-Nagel, Düren, Germany).

### 16S rRNA gene amplification and Illumina MiSeq sequencing

Vaginal lateral wall and endocervical swabs were collected for microbiome analysis by 16S rRNA gene sequencing. Matched lateral wall and endocervical swabs from the first sample visit were thawed and treated with an enzyme cocktail consisting of mutanolysin (25kU/ml, Sigma Aldrich, Modderfontein, RSA), lysozyme (450kU/ml, Sigma Aldrich), and lysostaphin (4kU, Sigma Aldrich) for 1 hour at 37 °C. This was followed by mechanical disruption using the Thermo Savant FastPrep 120 Cell Disrupter system for 3 × 30 seconds at speed setting 5.5 m/s. Microbial DNA was extracted using the PowerSoil DNA Isolation kit (Mo Bio Laboratories Inc., Germantown, MD, USA) following the manufacturer’s protocol. The V4 hypervariable region of the bacterial 16 S rRNA gene was amplified using modified universal primers^31^. Pooled duplicate samples were purified using Agencourt AMPure XP beads (Beckman Coulter, Brea, CA, United States) and quantified using the Qubit dsDNA HS Assay (Life Technologies, Carlsbad, CA, USA). Illumina sequencing adapters and dual-index barcodes were added to the purified amplicon products using limited cycle PCR and the Nextera XT Index Kit (Illumina, San Diego, CA, USA). Amplicons from 96 samples and controls were pooled in equimolar amounts and the resultant libraries purified by gel extraction (Qiagen, Hilden, Germany) and quantified. The libraries were sequenced on an Illumina MiSeq platform (300 bp paired-end) with v3 chemistry.

### 16S rRNA gene sequencing analysis

Following demultiplexing, raw reads were preprocessed as follows: forward and reverse reads were merged using usearch7^32^, allowing a maximum of three mismatches and the resultant merged reads were quality filtered using usearch7 (reads with E scores larger than 0.1 were discarded). Next, primer sequences were removed using a custom python script and the reads truncated at 250 bp. Sequences were then de-replicated whilst recording the level of replication for each sequence using usearch7. De-replicated sequences were sorted by abundance (highest to lowest) and clustered *de novo* into operational taxonomic units (OTUs) at 97% similarity using usearch7. Chimeric sequences were detected (against the Gold database) using UCHIME^33^ and removed. Individual sequences were assigned to the specific identifiers using a 97% similarity threshold. Taxonomic assignment was performed in QIIME 1.8.0^34^ using the RDP classifier (using the default confidence level of 0.5) against the GreenGenes 13.8 reference taxonomy for 97% identity. To increase species-level resolution, we used the usearch_global command implemented in VSEARCH^35^ to search the de novo picked OTUs’ representative sequences against a Custom Vaginal 16 S Reference Database (described previously^20^). All hits with ≥97% identity were accepted. The remaining OTUs (n = 109) were manually curated using both BLAST on NCBI’s nucleotide database (excluding uncultured organisms) and with usearch_global against the Vaginal 16S rDNA Reference Database^36^. Samples with ≥5000 reads were selected for downstream analyses. The OTU table was normalized (i.e. transformed to relative abundance * median sample read depth), and filtered to include OTUs with at least 10 counts in at least 20% of samples or a relative abundance of at least 0.001%.

### Statistical analyses

All downstream statistical analyses were performed in RStudio^37^ using the packages phyloseq^38^ for beta diversity analyses, metagenomeSeq^39^ for differential abundance testing, vegan^40^ for ordinations and redundancy analysis, and pheatmap^41^ and NMF^42^ for annotated heat maps. Microbiota subtypes were established by fuzzy clustering using the R package ‘cluster’^43^ with k = 3 (optimal k), a membership exponent of 1.25 and weighted Unifrac as the dissimilarity measure. Members with a probability of less than 60% of belonging to any of the three clusters were excluded from downstream analyses (n = 4).

Differences in study population characteristics were tested using the Chi-square, Fisher’s exact (when the expected value was < 5) or Mann-Whitney U test, accordingly. To examine whether the community type (C1–3) or any demographic or behavioral characteristic were associated with *C*. *trachomatis* infection we performed univariate and multivariate logistic regression analyses. Variation partitioning to investigate factors influencing microbial composition was performed by analysis of variance using distance matrices (adonis) with 999 permutations using weighted UniFrac distance measures. Specific differences in microbial composition between groups were assessed using metagenomeSeq’s MRfulltable function with a custom filter to determine significance. Merged taxa were deemed significantly different if they exhibited a fold change (beta coefficient) of ≥1.25, had an adjusted p-value of ≤0.05 and if at least one of the two groups being compared had ≥20% of samples with the given OTU/taxa OR the Fisher’s exact test result was significant (after multiple testing correction (MTC) by Benjamini-Hochberg method. OTUs were first merged at the lowest available taxonomic level using a custom script^20^. Random forests analyses were conducted on merged OTUs using the R package randomForest^44^. For randomForest, the data were randomly divided into training and test sets, comprising two thirds and one third of the data, respectively. Ecologic distances between lateral wall and endocervical samples from the same participants were compared with distances between samples from the same site from different particpants by comparing within-subject pair-wise distances (weighted Unifrac) with the mean of between-subject pair-wise distances using paired t-tests.

## Electronic supplementary material

Supplementary Figure
