# Supplementary material for: Endocervical and vaginal microbiota in South African adolescents with asymptomatic Chlamydia trachomatis infection
Source: Sci Rep. 2018 Jul 23;8:11109. doi: 10.1038/s41598-018-29320-x (PMC6056523; doi:10.1038/s41598-018-29320-x)
Supplement: Supplementary file 1 — Supplementary Figure [file 41598_2018_29320_MOESM1_ESM.docx]

**Endocervical and vaginal microbiota in South African adolescents with asymptomatic *Chlamydia trachomatis* infection**

Christina Balle, Katie Lennard, Smritee Dabee, Shaun L. Barnabas, Shameem Z. Jaumdally, Melanie A. Gasper, Venessa Maseko, Zizipho ZA. Mbulawa, Anna-Lise Williamson, Linda-Gail Bekker, David A. Lewis, Jo-Ann S Passmore, Heather B Jaspan

**Supplementary material**: Supplementary Figure 1.


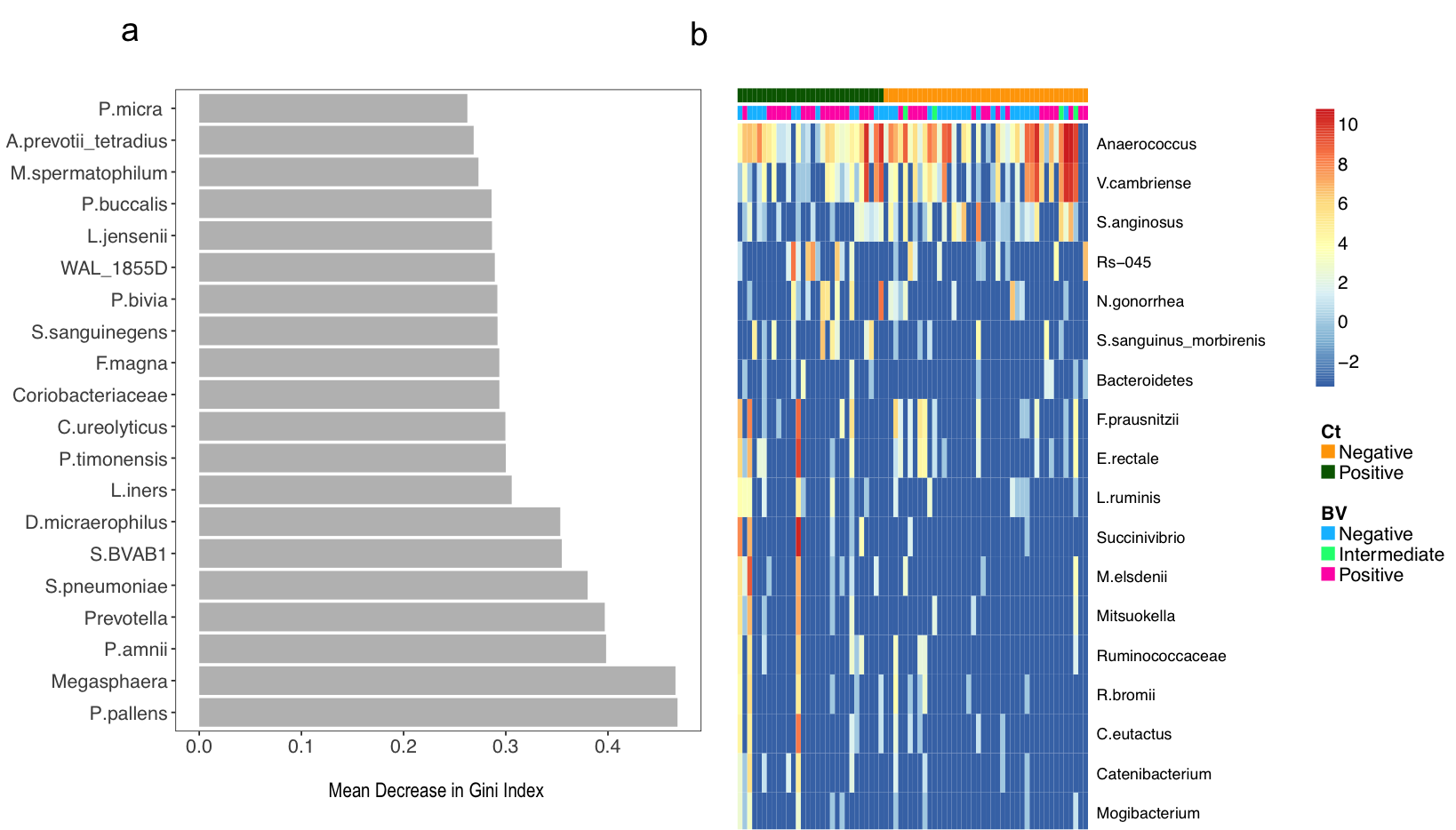


**Supplementary Figure 1**

1. The top 20 most influential taxa by Random Forest analysis. The x-axis indicates the mean decrease in Gini Index (length of bar represents predictive ability of each taxon).
2. Taxa significantly differentially abundant and/or frequent by *C. trachomatis* category in the vaginal lateral wall (LW) microbiota by metagenomeSeq (FDR ≤ 0.05, coefficient ≥ 1.25, taxa present in ≥ 20% of samples in at least one of the two groups being compared). Unsupervised clustering of samples (columns) by Bray-Curtis distance; heat map scale: log2-transformed standardized counts. The OTU assigned *C. trachomatis* taxonomy was excluded for this analysis.
